# Supplementary figures and images for: Genomic relatedness and dissemination of blaNDM-5 among Acinetobacter baumannii isolated from hospital environments and clinical specimens in Thailand
Source: PeerJ. 2023 Feb 7;11:e14831. doi: 10.7717/peerj.14831 (PMC9912941; doi:10.7717/peerj.14831)

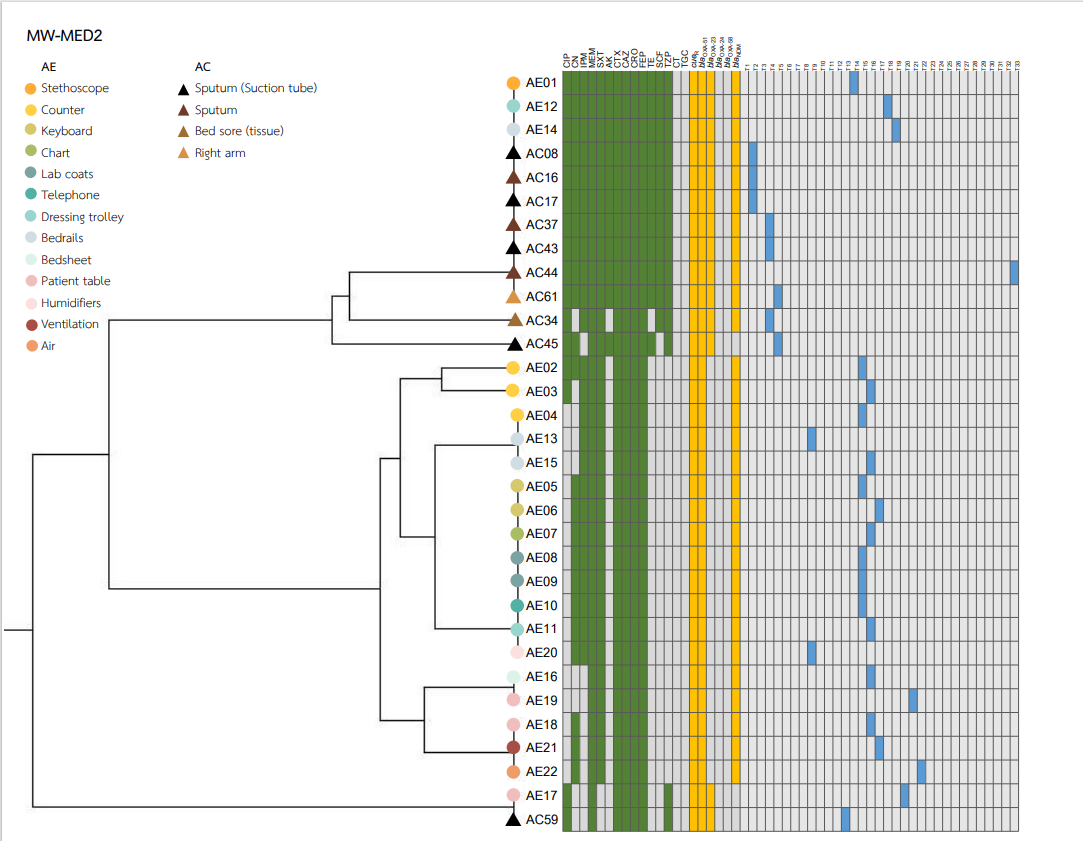

Supplement: Supplemental Information 6 [file peerj-11-14831-s006.png]

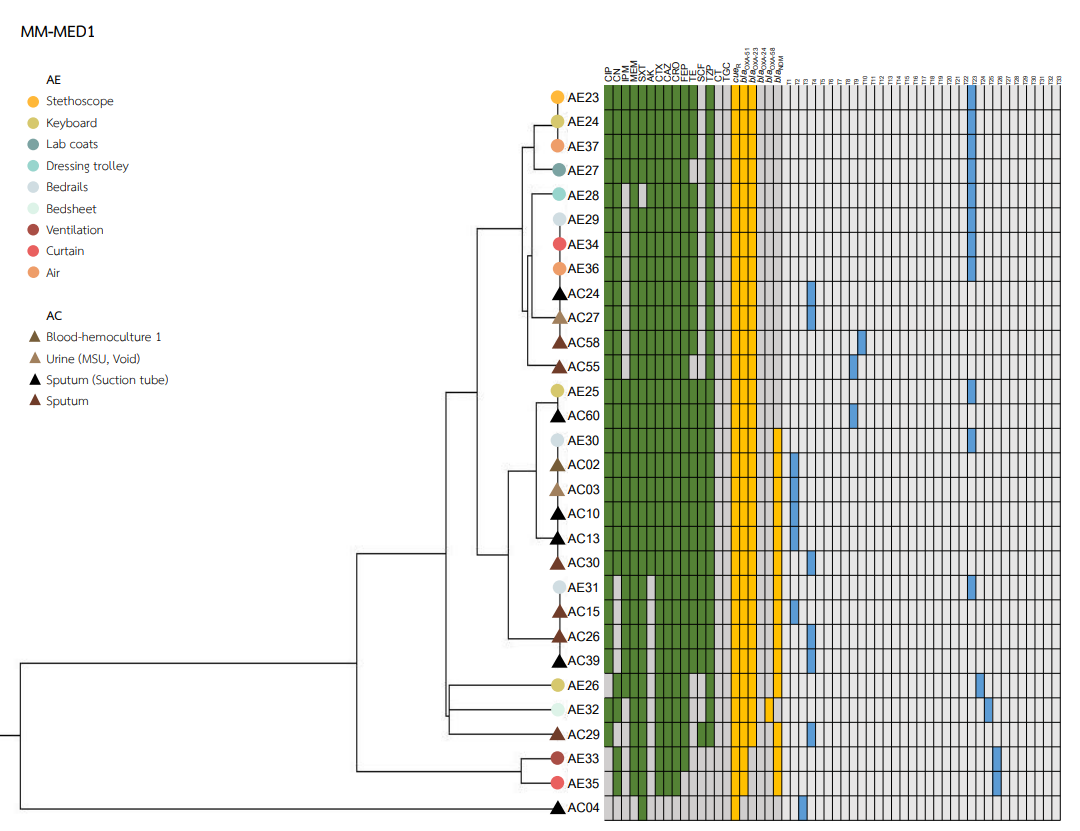

Supplement: Supplemental Information 7 [file peerj-11-14831-s007.png]

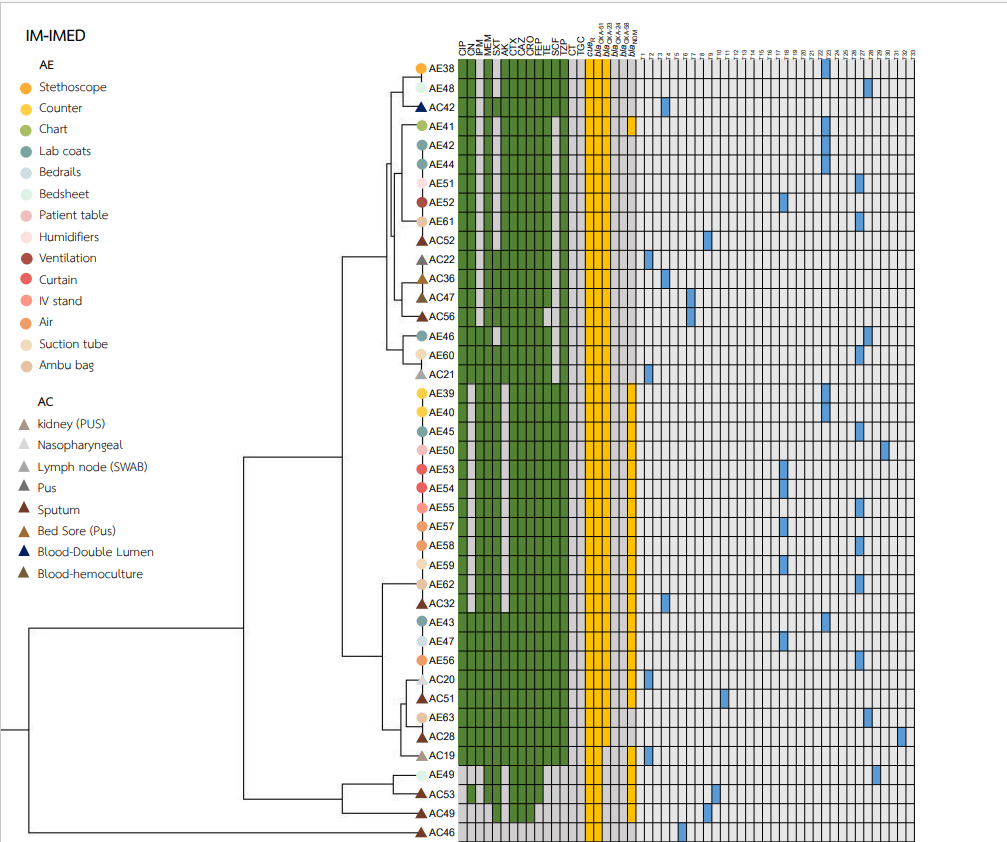

Supplement: Supplemental Information 8 [file peerj-11-14831-s008.png]

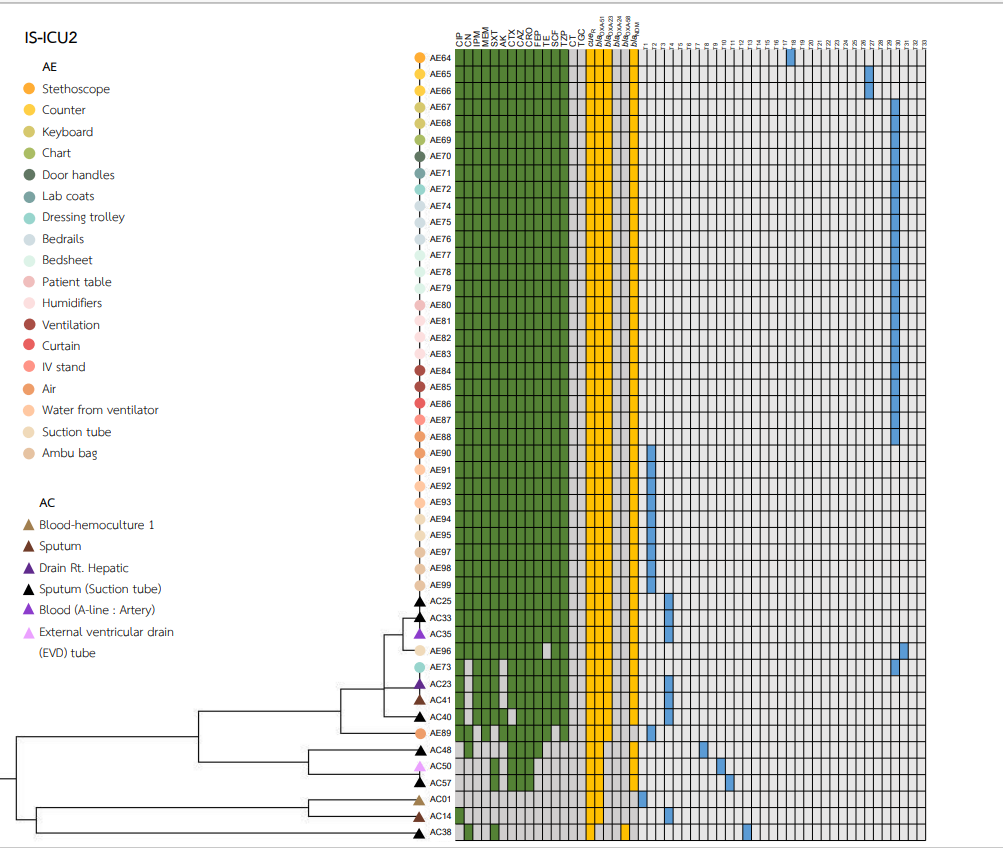

Supplement: Supplemental Information 9 [file peerj-11-14831-s009.png]

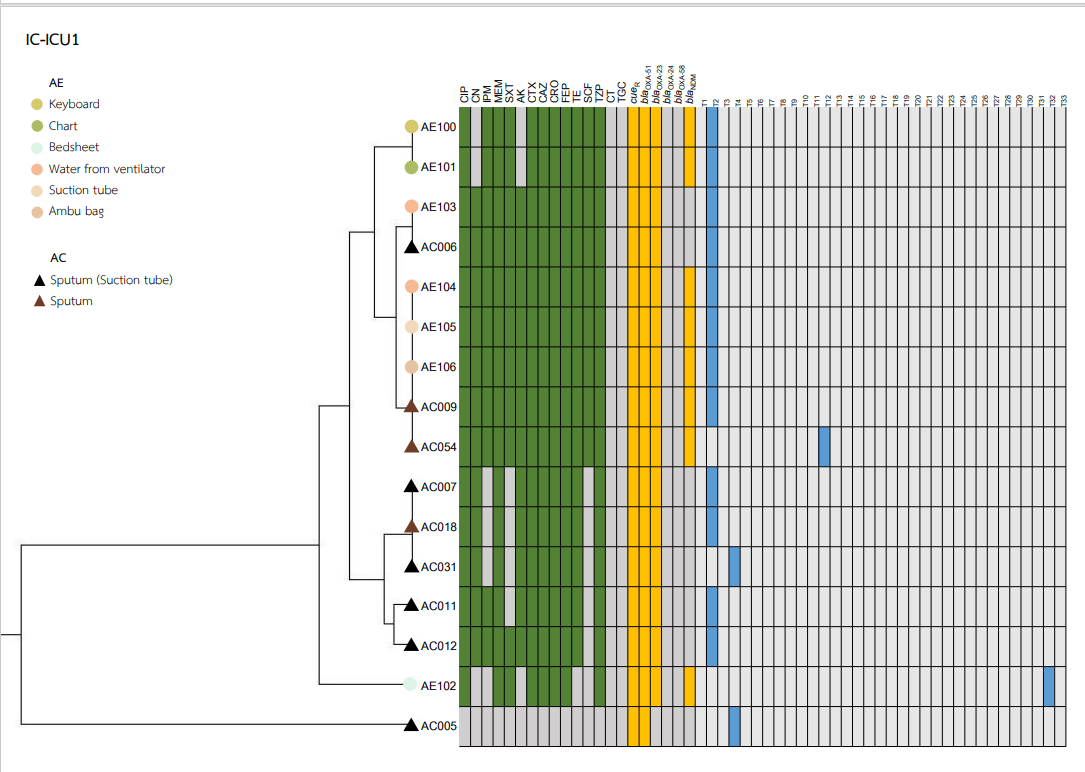

Supplement: Supplemental Information 10 [file peerj-11-14831-s010.png]
